# Editorial: Advances of health care transition for patients with childhood-onset chronic diseases: International perspectives, volume II

**DOI:** 10.3389/fped.2023.1147397

**Published:** 2023-02-13

**Authors:** Yuko Ishizaki, Ryota Ochiai, Mitsue Maru

**Affiliations:** ^1^ Department of Pediatrics, Kansai Medical University, Osaka, Japan; ^2^ Department of Nursing, Graduate School of Medicine, Yokohama City University, Yokohama, Japan; ^3^ School of Nursing, College of Nursing Art and Science, University of Hyogo, Akashi, Japan

**Keywords:** healthcare transition, childhood-onset chronic diseases, independence, transition readiness, social participation, family functioning

**Editorial on the Research Topic** Advances of health care transition for patients with childhood-onset chronic diseases: International perspectives, volume II

Owing to the advances in pediatric medicine, many children with chronic diseases are now able to survive till adulthood, often without serious sequelae or disabilities (1, 2). However, managing the health care of adult patients with childhood-onset chronic diseases (APCCD) is a challenge in contemporary pediatric practice; consequently, there is considerable discussion around the world regarding the state of health care transition for these patients.

In 1993, the American Academy of Adolescence and the American Academy of Pediatrics issued a joint statement on the medical care of adolescents with chronic conditions and defined transition as the “purposeful, planned movement of adolescents and young adults with chronic physical and medical conditions from child-centered to adult-oriented health care systems” (3). Earlier, a transition was to be accompanied by a voluntary transfer to adult-oriented care, meaning that the patient actively moves on their own will. Thirty years later, patients who find it difficult to become completely independent or are unable to make their decisions are also now eligible for transition and no longer require an accompanying obligatory transfer.

The “Six Core Elements of Health Care Transition™ 3.0” in the Got Transition, the federally funded national resource center on health care transition in the US, classified transition into three types: “transition youth to an adult health care clinician,” “transitioning to an adult approach to health care without changing clinicians,” and “integrating young adults into adult health care” (4). Since the concept of transition has evolved, we must recognize the transition of different patients in various fields.

Thus, the major issues regarding future transitions are proposed in this Research Topic. For patients who wish to transfer to an adult health care clinician, are independent, and are capable of social participation, future research must focus on institutional support for education and employment, factors associated with successful transition, and assessment of transition readiness.

Sakurai et al. performed a nationwide questionnaire-based survey on the prevalence and possible barriers to healthcare transition in Japan for APCCD in 2020. They stated that the top transitional barrier on the patient side were intellectual disability/rare disease and dependence on pediatrics, and lack of collaboration with adult healthcare was as medical/infrastructure factors; these problems seem to persist worldwide. Furthermore, Wakimizu et al. published a systematic review focusing on pediatric-to-adult healthcare transition interventions and promoting their effectiveness, and stated that enhanced interventions systematically support the transition, patient independence, and social participation.

Scarponi et al. reported on a particularly important part of their experience with the transition from pediatric-to-adult healthcare services for nephrological patients in Bologna–resistance from the hospital staff during the initial stage of transition to adult care. However, the difficulties caused by differences in pediatric and adult care models were resolved through participation in joint meetings and training sessions by both teams. For patients with cognitive deficits, they also contacted and established relationships with the local mental health facility in the patient's area of residence and ensured continuity between the hospital social services and those on the territory for families with social problems.

Biagioli et al. systematically reviewed the literature regarding self-care for improving the quality of life in children and young adults with chronic diseases. They found that both disease-specific and common instruments of self-care maintenance are being developed for various chronic diseases, with a special focus on treatment adherence. Instruments for self-care and self-monitoring of patients with chronic diseases may serve as important components to ensure a smooth transition as well as the success of a well-planned transition program.

Kobayashi et al. investigated the factors associated with the employment status and academic performance of childhood cancer survivors. Patients aged ≥18 years who participated in this single-center cohort study underwent comprehensive health check-ups for cognitive status, quality of life, transition readiness, and family function. The authors concluded that intellectual quotient, transition readiness, and family functioning were associated with employment status. Long-term follow-up of childhood cancer survivors should ensure comprehensive care to improve health, readiness to transition to self-care, and family functioning.

Interestingly, Takeuchi et al. conducted a narrative review of effective interventions to improve transition readiness in adolescents and young adults using the transition readiness assessment questionnaire, a widely used assessment tool for transition readiness. They identified 261 reports to extract and analyze three articles; all three interventions included were effective in improving transition readiness, particularly web-based and nurse-led organizational interventions.

There are two primary issues encountered in the recent transition-related research that require special mention. First, the question of what should be done about the transition of patients with severe physical and intellectual disabilities to adult care who cannot decide on the transition autonomously. Ishizaki et al. conducted a questionnaire-based survey of healthcare professionals involved in the care of adult patients with the 5p-syndrome, a chromosomal disorder with severe intellectual disability and various physical complications. Regarding support and welfare, all study participants had an experience of receiving consultation about care for the siblings of patients and only 15% of them believed the patient was on their way to the transition of patients with rare diseases and severe intellectual disabilities. This situation would be similar for other patients with severe disabilities due to chromosomal abnormalities.

Second, as a new topic, Kato et al. addressed the history behind the transition of heart transplant patients in Japan. They pointed out that paternalism in clinical settings that exists in Japan hinders the independence and decision-making process of the patients and their guardians. Although they describe it as a problem unique to Japan, the underlying ideas may be universal and the debate could be useful in addressing the problems faced by heart transplant patients across the globe.

There is a range of articles covering a broader perspective on this topic concerning patients with chronic diseases. We recommend that the ideal process of transition should be deliberated based not only on the diagnosis, but also on the patient's circumstances and characteristics, such as cognitive function, workability, and family function.
